# Carboplatin and Paclitaxel Chemoradiation for Localized Anal Cancer in Patients Not Eligible for Mitomycin and 5-Fluorouracil

**DOI:** 10.3390/cancers16173062

**Published:** 2024-09-03

**Authors:** Alyssa K. DeZeeuw, Michael F. Bassetti, Evie H. Carchman, Charles P. Heise, Dana Hayden, Elise H. Lawson, Cristina B. Sanger, Ray King, Noelle K. LoConte, Sam J. Lubner, Jeremy D. Kratz, Dustin A. Deming

**Affiliations:** 1 Division of Hematology, Medical Oncology, and Palliative Care, Department of Medicine, University of Wisconsin School of Medicine and Public Health, University of Wisconsin-Madison, Madison, WI 53792, USA; adezeeuw@wisc.edu (A.K.D.); ns3@medicine.wisc.edu (N.K.L.); sjlubner@medicine.wisc.edu (S.J.L.); jdkratz@medicine.wisc.edu (J.D.K.); 2 Carbone Cancer Center, University of Wisconsin, Madison, WI 53792, USA; bassetti@humonc.wisc.edu (M.F.B.); carchman@surgery.wisc.edu (E.H.C.); heise@surgery.wisc.edu (C.P.H.); haydend@surgery.wisc.edu (D.H.); lawson@surgery.wisc.edu (E.H.L.); sanger@surgery.wisc.edu (C.B.S.); kingray@surgery.wisc.edu (R.K.); 3 Department of Human Oncology, University of Wisconsin School of Medicine and Public Health, University of Wisconsin-Madison, Madison, WI 53792, USA; 4 Division of Colorectal Surgery, Department of Surgery, University of Wisconsin School of Medicine and Public Health, University of Wisconsin-Madison, Madison, WI 53792, USA; 5 William S Middleton Memorial Veterans Hospital, Madison, WI 53705, USA; 6 McArdle Laboratory for Cancer Research, Department of Oncology, Madison, WI 53705, USA

**Keywords:** anal cancer, carboplatin, paclitaxel, radiation, squamous cell carcinoma of the anus

## Abstract

**Simple Summary:**

Squamous cell carcinoma of the anus or anal cancer is increasing in prevalence, and the standard treatment of mitomycin and 5-fluorouracil is too toxic for many patients. Unfortunately, there is currently no standard of care for what to do for this disease when someone is not a candidate for this aggressive treatment. Here, we demonstrate the promising preliminary toxicity profile and efficacy of the combination of weekly carboplatin and paclitaxel chemotherapy for patients with localized anal cancer. This regimen was able to complete 80% of the intended treatments with anticipated toxicities, and 89% achieved a complete clinical response. This combination is a promising regimen and is already being investigated further in a clinical trial.

**Abstract:**

Background: Although squamous cell carcinoma of the anus (SCCA) is a relatively uncommon malignancy in the United States, it continues to increase in incidence. Treatment for locoregional disease includes mitomycin and 5-fluorouracil with radiation. This combination is associated with significant toxicity, limiting its use in patients who are older or have certain comorbidities. Carboplatin and paclitaxel (C/P) is an accepted treatment regimen for metastatic SCCA. We aim to evaluate the efficacy and toxicity of weekly C/P given with radiation for patients unable to receive standard chemoradiation for SCCA. Methods: From our cancer registry, adult patients who received weekly intravenous C/P concurrent with standard-dose radiation for localized SCCA were included in this study. Clinical response was determined based on the evidence of disease on imaging and/or anoscopy. Toxicities were graded according to the CTCAE v5. Results: Ten patients were included; eight were female, and the median age was 75.5 years (54–87). Six had T2 disease, and four had T3 tumors. Four had node-positive disease. The majority (70%) of patients were dosed at standard C (AUC 2) and P (50 mg/m^2^), with a limited subset requiring dose reduction for baseline performance status. Patients completed a mean of 78.3% (40–100%) of the intended treatments. A total of 89% of the patients achieved a complete clinical response. With a median follow-up of 25.8 months (3.4–50.4 months), 67% of the patients are alive and without recurrence. Two patients have had local recurrence, and one patient had metastatic progression. The most common toxicities of any grade included leukopenia (100%), anemia (100%), radiation dermatitis (100%), diarrhea (100%), and fatigue (100%). Grade 3 or higher toxicities included neutropenic fever (20%), neutropenia (30%), and anemia (30%). Conclusions: This study demonstrates promising tolerability and efficacy for weekly C/P chemoradiation for patients with anal cancer unable to receive mitomycin and 5-fluorouracil. This regimen merits further investigation in prospective clinical trials.

## 1. Introduction

Squamous cell carcinoma of the anus (SCCA) is relatively uncommon in comparison to other gastrointestinal tract malignancies, with an incidence of less than 10,000 cases per year in the United States [1]. Despite its low incidence, the rate of new SCCA cases has been increasing since the 1970s [2,3,4,5]. An analysis of SCCA between 2001 and 2015 revealed a 2.7% rise in incidence annually, which was most pronounced in individuals older than 50 years of age [4]. In addition to increased incidence, mortality associated with SCCA has also increased by 3.1% per year, with an especially prominent increase in older age groups [4]. These increases have remained highest in older women, regardless of race, and are associated with high rates of human papillomavirus (HPV) infection [4]. Similar trends have been observed more recently in a study published in 2023; an age-standardized incidence rate of 0.35 per 100,000 men and 0.57 per 100,000 women was reported, with most cases occurring in developed nations. An increase in the incidence of SCCA was also noted, with estimated global diagnoses increasing from 29,000 cases in 2018 to 30,000 in 2020 [5].

Given the increasing incidence of SCCA in older patients, treatment tolerance is a critical consideration. The current standard of care for locoregional disease involves upfront use of mitomycin and 5-fluorouracil (M/5FU) with concurrent radiotherapy, with the potential for cure, and without abdominoperineal resection and permanent ostomy, as opposed to upfront surgery [6,7,8]. M/5FU is associated with significant treatment-related toxicities which have led to the investigation of alternative chemotherapeutic regimens [9,10,11,12,13,14]. Specifically, a study published by Ajani et al. has shown that 87% of patients treated with mitomycin-based treatment experience at least one grade 3 or greater adverse event. In the same sample of patients, 61% had grade 3 or greater hematologic toxicity, and 74% had grade 3 or greater non-hematologic toxicity, such as fatigue, nausea, diarrhea, dermatological toxicity, and pulmonary toxicity [11]. Treatment-related mortality associated with M/5FU has been reported in 3–6.9% of patients treated with this regimen [15]. Additionally, long-term toxicity is problematic for patients after completing M/5FU chemoradiation [16,17]. Eliminating mitomycin from this regimen worsened disease-free survival at 4 years by 22%, although grade 4 toxicities were reduced by 16%, with similar response rates [18]. Similarly, the substitution of mitomycin with cisplatin resulted in inferior outcomes with a greater incidence of treatment failure (32.4% in the mitomycin-based treatment group versus 39.7% in the cisplatin-based treatment group), as evaluated in the RTOG 98-11, while still reporting significant adverse events [11]. Currently, there is no standard-of-care treatment option for localized SCCA when patients are not candidates for M/5FU chemoradiation.

In metastatic SCCA, a regimen of carboplatin (area under the curve [AUC] of 5 on day 1) and paclitaxel (80 mg/m^2^ on days 1, 8, and 15 on 28-day cycles) has become the first-line standard of care due to its efficacy and tolerability [6,19]. This regimen has a favorable response rate of 53% in the setting of metastatic disease [19]. Additionally, the InterAAct trial demonstrated that carboplatin and paclitaxel improve progression-free survival by 2.4 months and overall survival by 7.7 months when compared to cisplatin and fluorouracil [20]. This regimen also exhibited a more favorable toxicity profile than cisplatin and fluorouracil in this cohort of patients [20]. Common toxicities of carboplatin and paclitaxel include neutropenia (29%), infection (12%), fatigue (10%), and anemia (10%) [20].

This regimen has been found to be effective in other cancers. Carboplatin and paclitaxel are commonly used to treat squamous cell esophageal cancers in the localized setting [21,22]. Preoperative chemoradiotherapy according to the CROSS protocol consisting of intravenous carboplatin (AUC 2 mg/mL/min) and paclitaxel (50 mg/m^2^) on days 1, 8, 15, 22, and 29 was associated with improved median overall survival of 49.4 months in patients receiving chemoradiotherapy prior to surgery, compared to 24.0 months in patients having surgery alone [22]. This regimen also resulted in limited toxicity, with few patients having grade 3 or greater adverse events, with the most common being leukopenia (6%), anorexia (5%), fatigue (3%), and neutropenia (2%) [22].

The combination of carboplatin and paclitaxel has a favorable toxicity profile in combination with radiation, making this regimen an appealing alternative treatment option for SCCA. This treatment option could be particularly useful for patients with contraindications to mitomycin, such as those with immune deficiency, poor performance status, or cytopenias, for which providers may choose to use alternative regimens. Here, we describe the efficacy and tolerability of weekly carboplatin and paclitaxel chemoradiation in a cohort of patients with SCCA who did not receive M/5FU.

## 2. Materials and Methods

Subjects were consented as part of an Institutional Review Board-approved anal cancer registry at the University of Wisconsin Carbone Cancer Center. A retrospective chart review of the prospectively collected cancer registry was performed. Patients who received carboplatin and paclitaxel concurrent with radiation for SCCA between 1 July 2019 and 1 November 2023 were identified. The required criteria for inclusion were as follows: (1) pathologically confirmed squamous cell carcinoma of the anus, (2) locoregional disease without evidence of distant metastasis, (3) no history of pelvic radiation, and (4) received chemoradiotherapy with carboplatin and paclitaxel. The decision to treat each patient with carboplatin and paclitaxel was at each provider’s discretion. Ten patients met the criteria.

Pertinent data were obtained from medical records, including baseline demographics, duration and dosing of chemotherapy, radiation delivered, adverse events experienced from treatment initiation to two weeks after treatment completion, evidence of persistent disease, disease recurrence, and overall survival. All patients received treatment at the UWCCC, and no patients were lost to follow-up. Staging was established according to The American Joint Committee on Cancer (AJCC) *Cancer Staging Manual* 9th edition [23]. The response was determined based on clinical evidence of disease on imaging with positron emission tomography/computed tomography (PET/CT), surveillance anoscopy, and/or biopsy. All patients had a PET/CT within 3–8 months after completion of chemoradiotherapy. Follow-up imaging was performed with PET/CT or CT of the chest, abdomen, and pelvis yearly, or earlier if new symptoms arose. Surveillance anoscopy was performed in 6 patients starting between 2 and 14 months after the completion of chemoradiotherapy and was performed as often as every 3 months. The Common Terminology Criteria for Adverse Events (CTCAE) version 5.0 [24] was utilized to grade toxicities encountered. Statistical analysis was conducted using the Kaplan–Meier method to estimate overall survival and recurrence-free survival.

## 3. Results

### 3.1. Patient Baseline Characteristics

A total of 10 patients met the inclusion criteria and received weekly intravenous carboplatin and paclitaxel concurrent with standard-dose radiation for localized SCCA. Baseline characteristics are provided in Table 1. There were eight females and two males, with a median age of 75.5 years (range 54–87 years) at diagnosis. One patient was HIV-positive. Seven of these patients had cancer within the anal canal, while three patients had cancer of the anal margin. Six patients had T2 disease, and four patients had T3 tumors. None of the patients had T4 tumors. Six patients were without nodal involvement, and four patients met the criteria for N1a disease. The AJCC 9th edition staging varied between patients, with five having stage IIA disease, one patient with stage IIB disease, and four patients with stage IIIA disease. Multiple grades of disease were represented, with one patient having well to moderately differentiated disease, six patients with moderately differentiated disease, and one patient’s cancer being poorly differentiated. The remaining two patients had biopsy-proven invasive squamous cell carcinoma; however, differentiation status was not reported.

### 3.2. Completion of Intended Treatment

Chemotherapy doses of carboplatin ranged from an AUC of 1.5 to 2.0, with most patients receiving the intended dose of AUC 2.0, as detailed in Table 2. Paclitaxel doses ranged from 30 to 50 mg/m^2^, while radiation delivered was 50.4–58 Gy. Chemotherapy was given weekly. Patients completed a median of 85% of the intended treatment doses, and completed treatments ranged from 40 to 100% of the intended treatment doses. Three patients began treatment with a reduced dose of paclitaxel at 40 mg/m^2^, and one of these patients underwent further dose reduction to 30 mg/m^2^ paclitaxel and an AUC of 1.5 for carboplatin due to leukopenia, as well as poor tolerability and performance status. All patients completed radiation dosing as planned.

### 3.3. Toxicity Profiles

As demonstrated in Table 3, the most common adverse effects encountered were grade 1 diarrhea and fatigue, both of which all 10 patients experienced within the time period from treatment initiation to two weeks after treatment completion. Other common grade 1 toxicities included radiation dermatitis, perianal pain, thrombocytopenia, and nausea. The most frequently occurring grade 3 or greater toxicities were leukopenia, neutropenia, and anemia, each affecting three patients (30%). Two of the three patients meeting the criteria for grade 3 or greater neutropenia were admitted to the hospital for febrile neutropenia. One of these patients had a c. difficile infection. Two patients received only two of the anticipated five chemotherapy treatments due to poor tolerability, one patient received three of five treatments, and one patient received 4 of 5 days of treatment due to hematologic toxicities on one of the planned chemotherapy days.

### 3.4. Treatment Response and Efficacy

**Figure 1:**
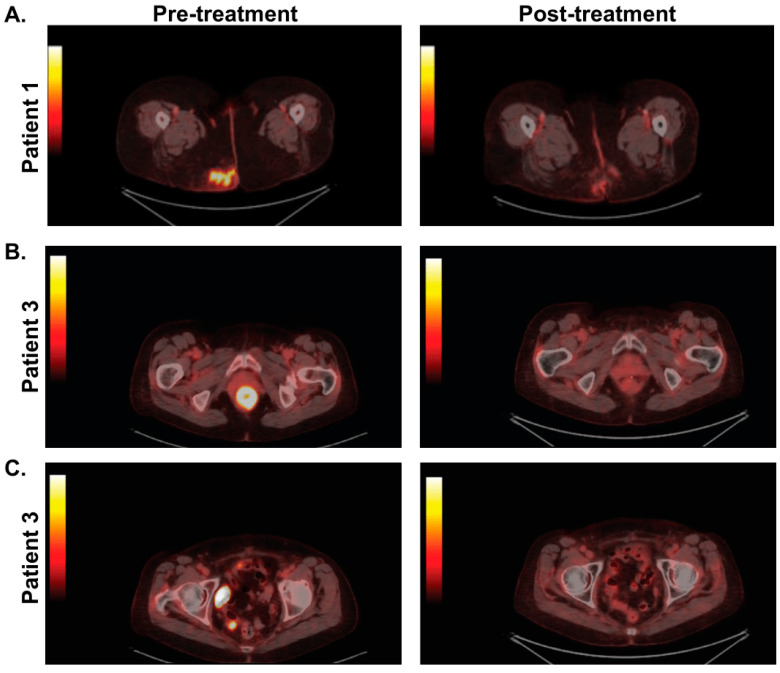
FDG PET/CT imaging pre- and post-treatment with carboplatin and paclitaxel chemoradiation. (**A**) Patient 1 had a squamous cell perianal cancer with gluteal extension pre-treatment and with resolution of the PET avidity in this area post-treatment. (**B**) Patient 3 had a highly PET-avid squamous cell carcinoma within the anal canal. (**C**) This patient also had extension to regional lymph nodes. The PET avidity resolved in both of these areas following chemoradiation.

Patients were followed for a median of 671 days (range 104–1532 days) after treatment initiation. Post-treatment surveillance consisted of PET/CT within 3–8 months of completion of chemoradiation followed by anoscopy with biopsy if examination or imaging findings were concerning for residual or recurrent cancer. Representative FDG PET/CT images from case 1, an SCCA of the anal margin, are presented in Figure 1A to demonstrate the reduction in fluorodeoxyglucose (FDG) avidity in the anus after treatment with carboplatin and paclitaxel chemoradiation in comparison to pre-treatment imaging. Additionally, pre- and post-treatment FDG PET/CT images are presented for patient 3 in Figure 1B,C who had an SCCA within the anal cancer with a nodal metastasis.

**Figure 2:**
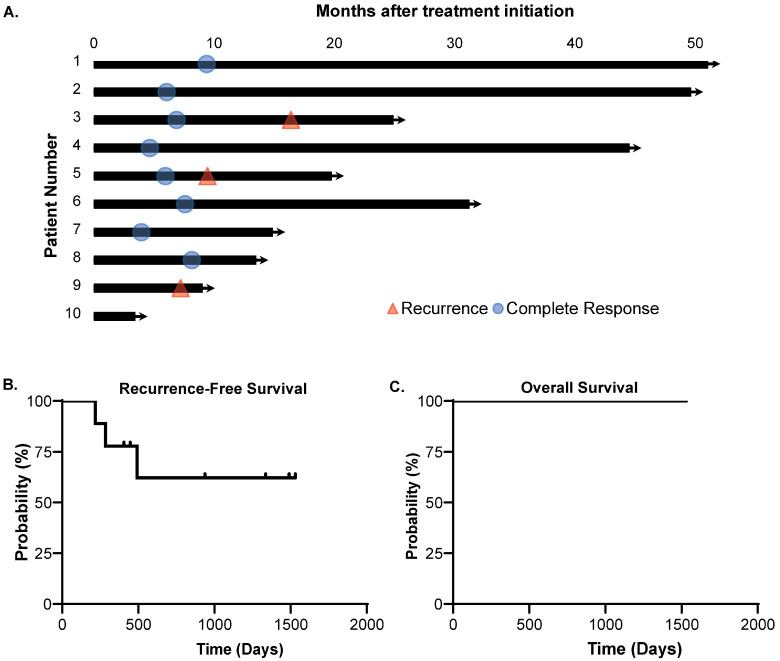
The clinical efficacy of carboplatin and paclitaxel chemoradiation for SCCA. (**A**) Swimmer’s plot demonstrating the clinical course of patients in this study. A complete clinical response was obtained in 8 of 9 patients where this assessment has occurred. (**A**,**B**) Three of the patients have developed recurrence. (**C**) All of the patients are still alive at the time of this analysis.

Nine of ten patients have completed treatment and have had repeat imaging and/or anoscopy. The one remaining patient has completed treatment and has no evidence of disease on physical examination, but has not yet had follow-up imaging or anoscopy to assess disease response. Of the nine patients that have been evaluated for response to treatment, eight (89%) have achieved a complete clinical response (Figure 2A). Of these eight patients achieving complete clinical response, 75% remain without cancer recurrence. Six of the nine patients (66.7%) evaluated for response to treatment have no evidence of recurrence (Figure 2B). Two patients have had local recurrence after achieving an initial complete clinical response, and one patient had metastatic progression of the disease without ever achieving a complete clinical response (Figure 2A,B). For the three patients with cancer recurrence, the mean time to recurrence was 331 days after treatment initiation (range 217–492 days). Notably, patients without recurrence received a greater percentage of the intended chemotherapy doses and cycles (86.6%) compared to patients who recurred, receiving 58.8% of the intended chemotherapy. All 10 patients included in this registry remain alive (Figure 2C). 

## 4. Discussion

With the changing demographic of anal cancer, it is becoming more common to identify patients with anal cancer who are not candidates for M/5FU-based chemoradiation. There is currently no standard of care for patients in this setting. Several trials have been conducted to identify alternate treatment options for non-metastatic SCCA. Unfortunately, most of these trials have resulted in inferior oncologic outcomes compared to treatment with M/5FU [11,18]. To identify effective therapeutic options for patients needing an alternative regimen, multiple clinical trials have investigated the use of radiotherapy alone or RT with a reduced dose of M/5FU. Radiotherapy alone has consistently demonstrated poorer outcomes compared to radiotherapy in combination with chemotherapy [25,26]. Reducing the dose of chemotherapy administered to older adults resulted in less severe toxicity; however, inferior oncologic outcomes relative to standard treatment have been reported [27]. There is a clear need for improved treatment options for SCCA, especially for patients ineligible for standard-of-care treatment. Given the sufficient evidence supporting the safety and efficacy of paclitaxel and carboplatin for metastatic SCCA [20] in conjunction with the findings presented here, the carboplatin and paclitaxel regimen shows promise for the treatment of localized anal carcinoma in the future.

In this retrospective study, we present 10 patients with SCCA who were not candidates for the standard-of-care treatment with M/5FU and were instead treated with weekly carboplatin and paclitaxel with concurrent radiation, similar to the CROSS protocol chemoradiation for esophageal carcinoma (22). Though this is a small cohort, the clinical complete response rate observed in this cohort is similar to that expected with stage-matched patients treated with M/5FU (89% versus 77–90%, respectively) [15,28] and better than historical cohorts of radiation alone (30–54%) [29,30]. This analysis demonstrated that carboplatin and paclitaxel chemoradiation may be an effective alternative regimen for the treatment of locoregional squamous cell carcinoma of the anus.

The toxicity rates in the patients treated with carboplatin and paclitaxel presented here are also comparatively lower than those reported in patients treated with M/5FU and radiation. Across these 10 patients treated with C/P and radiation, 40% had a grade 3 or greater adverse event compared to 71–87% of the patients treated with M/5FU and radiation experiencing a grade 3 or 4 adverse event [11,28]. The low-dose weekly schedule of C/P in this regimen lends itself to dose delays or reductions should patients develop toxicities. This aids in its safety profile while still allowing for substantial chemotherapy to be delivered over time should the patients tolerate it well.

One of the major limitations of this study is the presence of selection bias in the provider’s decisions regarding which patients are offered treatment with carboplatin and paclitaxel as opposed to the standard-of-care treatment with M/5FU. Other limitations of this study include the retrospective nature, small sample size, and lack of diversity in patient ages, sex, and demographics. The advanced age and low baseline functional status of patients in this cohort could also have impacted the tolerability of chemoradiation. Additionally, the follow-up time was short for many patients included. Despite these limitations, this study remains the first to examine the efficacy of carboplatin and paclitaxel with radiation for localized SCCA. This represents a reasonable option for patients with localized SCCA who are not candidates for M/5FU chemoradiation. This regimen is already being built upon in an on-going clinical trial evaluating the combination of carboplatin, paclitaxel, and pembrolizumab chemoradiation in this setting (NCT06493019).

In summary, treatment with carboplatin and paclitaxel given weekly along with radiation for localized anal cancer elicited a robust clinical response in a selected population with advanced age and comorbidities. This regimen is deserving of more extensive prospective trials with larger cohorts to better determine the tolerability and efficacy of the treatment and to compare its effectiveness against standard-of-care treatment.

## 5. Conclusions

Anal cancer continues to increase in incidence, and the standard treatment in the localized setting remains mitomycin and 5FU. This regimen has unacceptable toxicities for many patients, and de-escalation of this therapy is also likely appropriate for many early-stage patients. Currently, there is no established standard-of-care option for patients who are not candidates for mitomycin and 5FU. Here, we report the tolerability and efficacy of carboplatin and paclitaxel chemoradiation for the treatment of patients with localized anal cancer who were not deemed candidates for mitomycin and 5FU-based therapy. Overall, this regimen was tolerated in this cohort of patients. The weekly low-dose treatment strategy allows for early discontinuation and relatively quick recovery should toxicities become unacceptable. This regimen appears efficacious for these patients and should be considered an option for those who are not candidates for mitomycin and 5FU treatment. Additionally, this is a treatment backbone already being built upon in an on-going clinical trial.

## Figures and Tables

**Table 1:** Baseline patient characteristics.

Characteristics	Patients (*n* = 10)
**Age at diagnoses—years**	
Median	75.5
Mean	73.6
Range	54–87
**Sex—no. (%)**	
Male	2 (20)
Female	8 (80)
**ECOG Performance Score—no. (%)**	
1	9 (90)
2	1 (10)
**Tumor Location—no. (%)**	
Anal canal	7 (70)
Anal margin	3 (30)
**T Category—no. (%)**	
T2	6 (60)
T3	4 (40)
**N Category—no. (%)**	
N0	6 (60)
N1a	4 (40)
**AJCC Stage—no. (%)**	
IIA (T2N0)	5 (50)
IIB (T2N1a)	1 (10)
IIIA (T3N0)	1 (10)
IIIA (T3N1a)	3 (30)
**Histology—no. (%)**	
Squamous cell carcinoma	10 (100)
**Differentiation Status—no. (%)**	
Well to moderate	1 (10)
Moderate	6 (60)
Poor	1 (10)
Not reported	2 (20)
**HIV Positivity—no. (%)**	1 (10)
**P16 Status—no. (%)**	
Diffusely positive	4 (40)
Block-like positivity	2 (20)
Negative	1 (10)
Not evaluated	3 (30)

**Table 2:** Patient-specific staging and chemoradiotherapy dosing details.

Patient #	Age	Gender	Clinical TNM	Stage	Carboplatin Dose (# of Treatments)	Paclitaxel Dose (# of Treatments)	Total Radiation Delivered
1	63	Female	T2N0M0	IIA	AUC 2 (4)	50 mg/m^2^ (4)	54 Gy
2	76	Female	T2N0M0	IIA	AUC 2 (5)	50 mg/m^2^ (5)	54 Gy
3	81	Female	T3N1aM0	IIIA	AUC 2 (2), AUC 1.5 (3)	40 mg/m^2^ (2), 30 mg/m^2^ (3)	56 Gy
4	54	Male	T2N0M0	IIA	AUC 2 (5)	50 mg/m^2^ (5)	50.4 Gy
5	70	Female	T2N0M0	IIA	AUC 2 (2)	50 mg/m^2^ (2)	56 Gy
6	87	Female	T3N0M0	IIIA	AUC 2 (2)	40 mg/m^2^ (2)	58 Gy
7	75	Female	T2N0M0	IIA	AUC 2 (5)	50 mg/m^2^ (5)	54 Gy
8	79	Female	T2N1aM0	IIB	AUC 2 (5)	40 mg/m^2^ (5)	56 Gy
9	74	Male	T3N1aM0	IIIA	AUC 2 (3)	50 mg/m^2^ (3)	54 Gy
10	77	Female	T3N1aM0	IIIA	AUC 2 (5)	50 mg/m^2^ (5)	56 Gy

**Table 3:** Treatment-emergent adverse events encountered.

Toxicity Category	Grade 1	Grade 2	Grade ≥ 3
**Hematologic**			
Leukopenia	1 (10)	6 (60)	3 (30)
Neutropenia	2 (20)	1 (10)	3 (30)
Febrile neutropenia	0 (0)	0 (0)	2 (20)
Anemia	5 (50)	2 (20)	3 (30)
Thrombocytopenia	6 (60)	0 (0)	0 (0)
**Electrolyte abnormalities**			
Hypomagnesemia	0 (0)	2 (20)	0 (0)
Hypokalemia	0 (0)	1 (10)	0 (0)
**Gastrointestinal**			
Nausea	6 (60)	1 (10)	0 (0)
Diarrhea	10 (100)	0 (0)	0 (0)
Constipation	4 (40)	0 (0)	0 (0)
Abdominal pain	4 (40)	0 (0)	0 (0)
Oral mucositis	1 (10)	0 (0)	0 (0)
**Constitutional Symptoms**			
Fatigue	10 (100)	0 (0)	0 (0)
Dizziness	5 (50)	0 (0)	0 (0)
Anorexia	3 (30)	1 (10)	0 (0)
**Other**			
Perianal pain	8 (80)	0 (0)	0 (0)
Radiation dermatitis	9 (90)	1 (10)	0 (0)
Extremity edema	2 (20)	0 (0)	0 (0)
Paresthesia	1 (20)	0 (0)	0 (0)
Hypotension	0 (0)	1 (10)	0 (0)

## Data Availability

The data are contained within the article.
